# Blood oxygen regulation via P2Y12R expressed in the carotid body

**DOI:** 10.1186/s12931-024-02680-x

**Published:** 2024-01-27

**Authors:** András Iring, Mária Baranyi, Bernadett Iring-Varga, Paula Mut-Arbona, Zsuzsanna T. Gál, Dorina Nagy, László Hricisák, János Varga, Zoltán Benyó, Beáta Sperlágh

**Affiliations:** 1 Laboratory of Molecular Pharmacology, HUN-REN Institute of Experimental Medicine, Budapest, 1083 Hungary; 2 János Szentágothai School of Neurosciences, Semmelweis University School of PhD Studies, Budapest, 1085 Hungary; 3 Institute of Translational Medicine, Semmelweis University, Budapest, 1094 Hungary; 4 Cerebrovascular and Neurocognitive Disorders Research Group, Hungarian Research Network, Semmelweis University (HUN-REN-SU), Budapest, 1094 Hungary; 5 Department of Pulmonology, Faculty of Medicine, Semmelweis University, Budapest, 1083 Hungary

**Keywords:** Chemoreceptor, Hypoxia, O_2_ sensing, Purinergic signalling, Signal transduction, Cardiorespiratory regulation

## Abstract

**Background:**

Peripheral blood oxygen monitoring via chemoreceptors in the carotid body (CB) is an integral function of the autonomic cardiorespiratory regulation. The presence of the purinergic P2Y12 receptor (P2Y12R) has been implicated in CB; however, the exact role of the receptor in O_2_ sensing and signal transduction is unknown.

**Methods:**

The presence of P2Y12R was established by immunoblotting, RT qPCR and immunohistochemistry. Primary glomus cells were used to assess P2Y12R function during hypoxia and hypercapnia, where monoamines were measured by HPLC; calcium signal was recorded utilizing OGB-1 and N-STORM Super-Resolution System. Ingravescent hypoxia model was tested in anaesthetized mice of mixed gender and cardiorespiratory parameters were recorded in control and receptor-deficient or drug-treated experimental animals.

**Results:**

Initially, the expression of P2Y12R in adult murine CB was confirmed. Hypoxia induced a P2Y12R-dependent release of monoamine transmitters from isolated CB cells. Receptor activation with the endogenous ligand ADP promoted release of neurotransmitters under normoxic conditions, while blockade disrupted the amplitude and duration of the intracellular calcium concentration. In anaesthetised mice, blockade of P2Y12R expressed in the CB abrogated the initiation of compensatory cardiorespiratory changes in hypoxic environment, while centrally inhibited receptors (i.e. microglial receptors) or receptor-deficiency induced by platelet depletion had limited influence on the physiological adjustment to hypoxia.

**Conclusions:**

Peripheral P2Y12R inhibition interfere with the complex mechanisms of acute oxygen sensing by influencing the calcium signalling and the release of neurotransmitter molecules to evoke compensatory response to hypoxia. Prospectively, the irreversible blockade of glomic receptors by anti-platelet drugs targeting P2Y12Rs, propose a potential, formerly unrecognized side-effect to anti-platelet medications in patients with pulmonary morbidities.

**Supplementary Information:**

The online version contains supplementary material available at 10.1186/s12931-024-02680-x.

## Background

Cardiovascular diseases are one of the highest leading causes of death in the modern world [1]. Among the most common diseases leading to death are hypertension, heart failure, arrhythmias and myocardial infarction, as well as cardio-respiratory diseases such as sleep apnea [2]; morbidities that are closely regulated by the autonomic nervous system (ANS).

Compelling evidence has been shown for the tight interaction between the cardiac and respiratory systems mediated by the ANS, such as sinus arrhythmia under physiological conditions; however, cardiorespiratory interactions become notably evident under stress conditions such as hypoxia and hypercapnia (H/H). While hypoxia affects primarily peripheral chemoreceptors in the glomus caroticum (carotid body, CB) and the aortic bodies, hypercapnia acts mainly through chemoreceptors in the brainstem [3]; severely influencing respiratory rate, vagal and sympathetic nerve traffic and consequently modulating the heart rate and blood pressure [4, 5]. Similarly, CB denervation results in the absence of normocapnic ventilatory responses to hypoxia and reduced respiratory responses to hypercapnia [6]. Interestingly, however, the precise mechanism of how the CB senses hypoxia is largely unclear.

Structurally, the CB contains type I cells, expressing tyrosine hydroxylase enzyme, and type II cells, positive for glial fibrillary acidic protein [7]. Type I cells are recognized as the chemoreceptor element that produces neurotransmitters and peptide neuromodulators; while type II cells (or sustentacular cells) envelop clusters of type I cells, and are believed to have a supportive role, although might function as stem cell precursors for type I cells [8]. Neurotransmitters and -modulators released during chemoreflex activation from type I cells have been reported to consist primarily of ATP, adenosine, acetylcholine, dopamine, noradrenaline, serotonin, glutamate, GABA, and histamine [9–13].

Changes in the blood O_2_ tension, which is one of the primary signal of hypoxic chemotransduction, evoke changes in the mitochondrial membrane potential and NADH levels, consequently modulating ion-channel function (e.g. TASK1, TASK3, BK channels [14]), cell membrane-depolarization and extracellular calcium influx [15]. However, recent advances in the chemosensory functions of the CB proposed that glomus cells are in fact multimodal sensors [16].

G-protein coupled receptors (GPCRs) expressed in glomus cells have been intensively investigated, since these receptors are ideal candidates for pharmacological interventions [17]. Utilizing RNA sequencing of the CB biased towards GPCRs and comparing the expression profile with that of the adrenal medulla, which shares developmental similarities with the CB, olfactory receptor 78 (Olfr78) was identified as a de facto hypoxia sensor [18]. Proposedly, when blood oxygen concentration decline, lactate accumulation occurs, which activates Olfr78 and modulates the release of the neurotransmitter dopamine to regulate carotid sinus nerve activity [18]. However, a recent study suggested that lactate sensing may preferentially occur by rapid transport into glomus cells, inducing an increase in the cytosolic NADH/NAD^+^ ratio, activating membrane cation channels, and leading to cell depolarization [19]; further corroborating that hypoxia sensing requires the interplay of multiple signal modalities [20].

Single cell transcriptome analysis of young, healthy mouse CB glomus cells identified several highly expressed GPCRs, most interestingly P2Y purinoceptor 12 (P2Y12R) and Cannabinoid receptor 1 (CB1R) [21]. Previously, preliminary findings by Agarwal and colleagues indicated the presence of P2Y12R protein on cells isolated from CB and report on the function of P2Y12R inhibitors on intracellular calcium changes; however, their exploratory results have not been published or validated [22].

In the present work, we have intended to elucidate the involvement of P2Y12R and CB1R in the peripheral oxygen sensing and signal transduction mechanism; and to evaluate the influence of the receptors on the cardiorespiratory compensatory response during ingravescent hypoxia.

## Materials and methods

### Animals

All procedures involving animal care and use in this study were performed using wild-type (C57/Bl6N) and *P2ry12* gene deficient (*P2ry12*^*–/–*^) or littermate control (*P2ry12*^+*/*+^) mice of mixed gender, aged 8–14 weeks, with an average weight of 30 ± 4.0 g. The original breeding pairs of *P2ry12*^*–/–*^ knockout mice, B6;129-P2ry12^tm1Dgen^/H were obtained from Deltagen Inc. (San Matteo, CA, USA). Cloning and breeding strategy, and genotyping protocol have been described previously [23]. All mice were backcrossed onto a C57BL/6N background at least 8 to 10 times, and experiments involving the knock-out strain were performed with littermates as controls. Animals were housed under a 12-h light–dark cycle in a temperature- (23 ± 2 °C) and humidity-controlled room (60 ± 10%) and had access to food (ssniff^®^ Souris-Elevage E, 10 mm pellet, Cat# S8189-S096; ssniff Spezialdiäten GmbH, Soest, Germany) and water ad libitum. All studies in vivo were carried out during the light phase of the cycle. Maximum of three experimental animals were kept in a standard cage with bedding material of corncob. Cardboard bedding materials and tubes were applied to all the cages for environmental enrichment. Experimental animals were randomly assigned to experimental groups prior to the start of the experiment. Data acquisition and evaluation were performed by investigators blind to the experimental status of the subject.

### Materials

Adenosine 5’-diphosphate (Cat. No. A2754; PubChem CID: 12797869), bovine serum albumin (Cat. No. A2153), Calcium Ionophore A-23187 (Cat. No. C7522; PubChem CID: 40486), Collagenase from Clostridium histolyticum (Cat. No. C0130), Trypsine (Cat. No. T9935), Elastase from porcine pancreas (Cat. No. 45125), Insuline solution from bovine pancreas (Cat. No. I0516), Penicillin–Streptomycin (Cat. No. P4333; PubChem CID: 78174239), DMEM/F-12 with HEPES and L-Glutamine (Cat. No. DF-041), Calcium chloride dihydrate (Cat. No. C3306; PubChem CID: 6093260), 6-hydroxydopamine hydrochloride (Cat. No. H4381; PubChem CID: 160157), Atipamezole (Cat. No. A9611; PubChem CID: 71310) and Poly-L-Lysine (Cat. No. P9155) were purchased from Sigma-Aldrich. ARL 67156 (Cat. No. 1283; PubChem CID: 11957464), Clopidogrel hydrogen sulfate (Cat. No. 1820; PubChem CID: 115366) and PSB 0739 (Cat. No. 3983; PubChem CID: 44583582) were from Tocris Biosciences, Bristol, UK. Ketamine (Calypsol, Reg. No. OGYI-T-3609/01; PubChem CID: 15851) was from Richter Gedeon Plc., Budapest, Hungary, and Xylazine (CP-Xylazine, Cat. No. 1206; PubChem CID: 68554) was from C.P. Pharma Handelsgesellschaft mbH, Burgdorf, Germany. Oregon Green^™^ 488 BAPTA-1, AM (OGB-1, Cat. No. O6807) was from Invitrogen (Thermo Fisher Scientific, Waltham, MA, USA). Antibodies directed against mouse thrombocytes were from BD Biosciences, Franklin Lakes, NJ, USA (Purified Rat Anti-Mouse CD41, Cat. No. 553847, RRID:AB_395084), against tyrosine hydroxylase was from Sigma-Aldrich (Cat. No. AB152; Lot. No. 3256647, RRID:AB_390204), against P2Y12 receptor was from AnaSpec, Fremont, CA, USA (Cat. No. AS 55043A; Lot. No. UB1701, RRID:AB_2298886), against β-actin was from Cell Signaling Technology, Danvers, MA, USA (Cat. No. 4967; Lot. No. 19, RRID:AB_330288), and against CB1 receptor was a kind gift from Dr. István Katona and Dr. Zsolt Lele, and was custom produced by Immunogenes Kft., Budakeszi, Hungary, and was originally validated and described in [24].

### Expression analysis

Total RNA was isolated using the RNeasy Micro Kit (Qiagen) with the additional step of on-column DNA digestion according to the manufacturer’s instructions. Quality control of samples was carried out using a Nanodrop 2000c Spectrophotometer (Thermo Fisher Scientific, Waltham, MA, USA). Obtained RNA was immediately reverse-transcribed using the SuperScript^™^ IV VILO^™^ cDNA synthesis kit (Thermo Fisher Scientific, Waltham, MA, USA) according to the manufacturer′s instructions and samples were stored on −20 °C until further assay was performed. Reverse transcriptase PCR reactions were carried out using TaqMan™ Fast Advanced Master Mix (Thermo Fisher Scientific, Waltham, MA, USA) according to the manufacturer′s instructions. Primers for murine GAPDH (Mm99999915_g1), CB1R (Mm01212171_s1) and P2Y12R (Mm01950543_s1) were designed, validated and synthesized by Thermo Fisher Scientific Inc. (Waltham, MA, USA), and quantification was performed using the Applied Biosystems ViiA 7 Real-Time PCR System (Thermo Fisher Scientific Inc., Waltham, MA, USA). Relative expression levels were obtained after normalization with GAPDH values to account for intra-well variability.

### Western blotting

Cells were lysed in radioimmunoprecipitation assay (RIPA) buffer containing 150 mM NaCl, 50 mM Tris–HCl (pH 7.4), 5 mM EDTA, 0.1% (w/v) SDS, 0.5% sodium deoxycholate and 1% Triton X-100 as well as protease inhibitors (10 mg/ml leupeptin, pepstatin A, 4-(2-aminoethyl) benzensulfonyl-fluorid and aprotinin) and phosphatase inhibitors (PhosSTOP™, Roche AG, Basel, Switzerland). Total cell lysates were separated by sodium dodecyl sulfate–polyacrylamide gel electrophoresis. Protein was then transferred onto nitrocellulose membranes, followed by overnight incubation with primary antibodies. Membranes were incubated with horseradish peroxidase-conjugated secondary antibodies (Cell Signaling Technology Inc., Danvers, MA, USA) for one hour at room temperature and were developed using the ECL detection system (Thermo Scientific Pierce, Life Technologies, Waltham, MA, USA). After evaluation, antibody dissociation from the membrane was induced using Restore™PLUS Western Blot Stripping Buffer (ThermoFisher Scientific, Waltham, MA, USA) according to the manufacturer’s instructions, and membranes were then reprobed with antibodies recognizing the β-actin protein. Protein band intensities were analyzed by ImageJ software (NIH). Intensity values of bands representing specific proteins were normalized to the intensity of the band representing β-actin.

### Immunohistochemical analyses

Mice were euthanized by gradually filling the chamber, with a displacement rate of approximately 30% to 70% of the chamber volume/min with CO_2_ where aversion or distress was minimized, and the chest cavity was opened for perfusion with 4 °C phosphate buffered saline (PBS) followed by fixation with 4% PFA at room temperature for 20 min. After fixation, common carotid arteries were carefully removed *en bloc*, and fixed samples were cryoprotected in optimal cutting temperature compound (Tissue-Tek® O.C.T. Compound, Sakura Finetek USA Inc., Torrance, CA, USA) using an isopentane-dry ice slurry. Subsequently, the sample was sectioned with a cryostat microtome (Thermo Shandon Cryotome, ThermoFisher Scientific, Waltham, MA, USA) at 10 μm thickness. Samples were washed three times with PBS and incubated in PBS containing 5% BSA and 0.3% Triton X-100 for 2 h. Samples were randomly divided into groups and were incubated overnight at 4 °C in the same buffer containing anti-Tyrosine Hydroxylase antibody (1:400), together with anti-P2Y12R antibody (1:1000) or anti-CB1R antibody (1:500). The specificity of the anti-P2Y12-receptor antibody has been validated in samples from wild-type control and P2Y12R-KO mice previously [25]. After overnight incubation, samples were washed in PBS and incubated with the appropriate AlexaFluor-564 conjugated antibody and AlexaFluor-488 conjugated secondary antibody (Molecular Probes, Thermo Fisher Scientific, Waltham, MA, USA; 1:200) as well as with Hoechst 33342 (Thermo Fisher Scientific, Waltham, MA, USA; 1:10000) for 1 h avoiding exposure to light. For cell culture experiments, primary murine glomus cells were isolated and cultured on cover slips placed in cell culture dishes. To visualize protein expression, cells were washed with ice-cold PBS, thereafter fixed in 4% PFA at room temperature for 20 min. Following, cells were washed three times with PBS and incubated in PBS containing 5% BSA and 0.3% Triton X-100 for 2 h. Samples were incubated overnight at 4 °C in the same buffer containing anti-Tyrosine Hydroxylase antibody (1:400), together with anti-P2Y12R antibody (1:1000) or anti-CB1R antibody (1:500). After overnight incubation, samples were washed in PBS and incubated with the appropriate AlexaFluor-564 conjugated antibody and AlexaFluor-488 conjugated secondary antibody (Molecular Probes, Thermo Fisher Scientific, Waltham, MA, USA; 1:200) as well as with Hoechst 33342 (Thermo Fisher Scientific, Waltham, MA, USA; 1:10000) for 1 h avoiding exposure to light. Samples were mounted in ProLong^™^ Gold Antifade Mountant (Thermo Fisher Scientific, Waltham, MA, USA) overnight. Immunofluorescent signal was analyzed using a Nikon Eclipse Ti-E inverted microscope (Nikon Instruments Europe B.V., Amsterdam, The Netherlands), and a C2 laser confocal system. Immunofluorescent signal intensity was quantified following the protocol of Shihan et al. [26]. Briefly, during image acquisition, identical laser settings for gain, offset and intensity parameters were used. The acquired pictures were processed in ImageJ Fiji, where initially the background was subtracted for all channels (rolling ball radius: 50 pixels), followed by the automated measurement of mean fluorescent intensity for each channel. Quantitative analysis of immunostaining was performed on at least three, randomly selected fields within the region of interest for each sample. Three independent samples were analysed from each animal.

### Isolation of glomus cells

Isolation of primary murine glomus cells was modified after Ortega-Sáenz et al. [14], and performed as follows: experimental animals were sacrificed and the carotid bifurcation on both sides was harvested (two per enzymatic reaction). Tissues were placed in 1 ml PBS, supplemented with 0.6 mg collagenase II, 0.3 mg trypsin, 10 U/mL Porcine elastase and 50 µM CaCl_2_ for 20 min at 37 °C. Subsequently, tissue samples were teased apart and incubated for another 5 min. Dissociated cells were plated on poly-L-lysine coated dishes and culture medium (DMEM/F-12 (Dulbecco’s modified Eagle’s medium) with 10% fetal bovine serum, 1% penicillin/streptomicin, 1% L-glutamine, and 84 μU of insulin per ml) was added. Cells were allowed 24 h to adhere, and left to grow for 7 days, where medium was changed daily.

### Controlled in vitro hypoxia and hypercapnia

Cytation 5 Cell Imaging Multi-Mode Reader (Agilent Technologies Inc., Santa Clara, CA, USA) equipped with O_2_/CO_2_ gas controllers was used to induce mild (12% O_2_/5% CO_2_/83% N_2_), moderate (6% O_2_/10% CO_2_/84% N_2_) or severe hypoxia / hypercapnia (1% O_2_/15% CO_2_/84% N_2_) levels at 37 °C. Glomus cells were grown in 24-well plates and were placed into the reading chamber of the instrument for 60 min with the lids on to minimize the evaporation of the culture medium. Samples of each hypoxia/hypercapnia step were taken and replaced with the same amount of fresh medium prewarmed at 37 °C. Finally, the calcium-ionophore, A-23187 (1 μM, 1 min) was added to the medium, in order to evaluate the exocytotic capacity of the remaining monoamines stored in vesicles in the glomus cells. At the end of the experiment, cells were lysed in ice-cold 0.01 M perchloric acid solution and were collected for protein measurement [27].

### Mono- and catecholamine measurement by HPLC analysis

Catechol- and indole amines, nucleotides (ATP, ADP, AMP) and adenosine were determined using HPLC method. Isolated primary glomus cells obtained from wild-type control or P2Y12R-KO animals were pretreated with PSB 0739 (500 nM, 30 min) or the vehicle and 250 µl supernatant samples before and after stimulation with ADP (10 μM, 5 min) were collected in 50 µl of ice-cold 0.01 M perchloric acid containing theophylline (as an internal standard) at a concentration of 10 μM and 0.5 mM sodium metabisulfite (as an antioxidant), termed as “extraction solution”. Subsequently, the calcium-ionophore, A-23187 (1 μM, 1 min) was added to the medium, in order to evaluate the exocytotic capacity of the remaining monoamines stored in vesicles in the glomus cells. At the end of the experiment cells were collected in 100 µl “extraction solution”, disrupted by sonication and centrifuged at 3510 × g for 10 min at 4 °C. Protein content was measured according to Lowry et al. [28]. To validate the adequacy of the peripheral chemical sympathetic denervation, carotid body tissue and blood plasma were collected after 6-OHDA treatment. CBs were carefully removed *en bloc* and placed in liquid nitrogen and homogenized by sonication in 200 µl of ice-cold “extraction solution”. The protein of the tissue extract and the neutralizing potassium perchlorate precipitate were removed by centrifugation at 3510 × g for 10 min at 4 °C. The clean supernatant was stored at −20 °C until analysis, the first pellet was used to measure the protein content. For plasma measurements, blood samples were collected by venous puncture in pre-cooled heparin-coated vials, and then carefully centrifuged at 2200 × g for 10 min at 0 °C. The platelets and the remaining cells were removed by repeated centrifugation (6800 × g for 5 min at 0 °C) and 100 µl plasma samples were treated with 10 µl of ice-cold 4 M perchloric acid, which contained the internal standard and antioxidant at a concentration 10 × higher than the “extraction solution”. Perchloric anion from the samples was precipitated by 4 M K_2_HPO_4_, and the centrifugation step was repeated. Sample extracts were stored at −20 °C until analysis.

Quantification of nucleotides and biogenic amines from tissue was performed by online column switching separation. ACE Ultra Core Super 5 μm particle size packed columns from A.C.T.L. (Scotland) were used for analysis. Solid phase extraction (SPE) was carried out on a Phenyl-Hexyl packed (7.5 cm × 2.1 mm) column and for separation it was coupled to the C-18 (150 × 2.1 mm) analytical column. The flow rate of the mobile phases [“A” 10 mM potassium phosphate, 0.25 mM EDTA “B” with 0.45 mM octane sulphonyl acid sodium salt, 8% acetonitrile (v/v), 2% methanol (v/v), pH 5.2] was 350 or 450 µl/min, respectively in a step gradient application [29]. Enrichment and stripping were performed with the [10 mM potassium phosphate, pH 5.2] buffer for 4 min at a flow rate of 300 µl/min, the separation time was 55 min. Shimadzu LC-20 AD HPLC system was used for analysis. The analytes were signaled with Agilent UV. (1100 series variable wavelength) and a (BAS CC-4) amperometric detectors in cascade mode. Monoamines were detected electrochemically at an oxidation potential of 0.73 V, while the internal standard, the nucleotides and adenosine were signaled by UV. at 253 nm. Concentrations were calculated by a two-point calibration curve internal standard method: (Ai × f × B)/(C × Di × E) (Ai: Area of nucleotide or biogenic amine component; B: Sample volume; C: Injection volume; Di: Response factor of 1 pmol biogenic amine or 1 nmol nucleotide standard; E: Protein content of sample; f: factor of Internal Standard (IS area in calibration / IS area in actual)). Data was expressed as pmol / mg protein, unless stated otherwise.

### Calcium measurement

For the functional study of glomus cell activity, primary cells were incubated for thirty minutes with 1 µM pluronic acid (F-127 P3000MP, Sigma-Aldrich, Cat. No. P2443, previously warmed at 37 °C) diluted 1:1 with 5 µM Oregon Green 488 BAPTA-1 (OGB-1, Thermo Fisher Scientific, Waltham, MA, USA, Cat. No. O6807) in 2 mL of the culture medium (DMEM/F-12 with 10% fetal bovine serum, 1% penicillin/streptomicin, 1% L-glutamine, and 84 μU of insulin per ml). Following incubation, the medium with the dye was replaced with fresh medium and cells were incubated for an additional twenty minutes. Spontaneous activity of the cells was recorded for five minutes using N-STORM Super-Resolution System at 38% intensity of the channel, at 30 ms/Hz, 12-bit (no binning) without delay between frames at 20 × magnification. Following the registration of the baseline activity, cells were stimulated with the acute administration of adenosine 5′-diphosphate (ADP, 10 µM) for eight minutes. As a control, the calcium ionophore A-23187 (1 µM) was applied, and fluorescence changes (ΔF/F) were calculated per each individual cell.

### Drug administration

Clopidogrel ((S)-( +)-Methyl 2-(4,5,6,7-tetrahydrothieno[3,2-c]pyridin-5-yl)-2-(2-chlorophenyl) acetate hydrogen sulfate, diluted in physiological saline for injection) and its vehicle (physiological saline) were administered intraperitoneally (10 mg/kg in 150 μl per mouse, 60 min prior to the experiment); PSB 0739 (dissolved in physiological saline) or its vehicle were administered intrathecally (0.3 mg/kg in 5 μl per mouse, 18 h prior to the experiment), where experimental animals were anesthetized with 2% isoflurane (in air with 0.8 L/min flow rate using precision anesthetic vaporizers). All drug solutions were freshly prepared on the day of use. The administration of a drug to the cerebrospinal fluid surrounding the spinal cord is known as intrathecal administration [30]. Intrathecal administration enables the direct administration of small molecules that are otherwise unable to cross the blood–brain barrier to the central nervous system without damaging the spinal cord [31]. Previous studies showed that PSB 0739 can hardly penetrate the blood brain barrier due to its chemical character [32], therefore this route of drug administration allowed for the selective targeting of the centrally expressed P2Y12R (i.e. expressed on microglia), without influencing the peripherally expressed receptors. The efficiency of the intrathecal delivery route was verified by HPLC analysis of PSB 0739 presence in the rostral spinal cord, trigeminal nucleus caudalis (TNC), somatosensory cortex (S1) and prefrontal cortex (PFC) in our previous publication [33]. 6-OHDA hydrochloride was administered to achieve peripheral chemical sympathetic denervation. To counteract the potential adverse effects of the sympathetic denervation, such as the marked reduction in blood pressure and heart rate, a modified treatment protocol was used as described by Soto-Piña et al.: 20 mg/kg 6-OHDA diluted in 0.9% saline and 0.1% ascorbic acid were injected intraperitoneally for six alternative days. The control group received injections of vehicle (saline and ascorbic acid) SPS:refid::bib34(34).

### Platelet depletion

The mouse-specific anti-CD41 antibody was applied to deplete thrombocytes, as described previously [33, 35]. Briefly, wild-type control mice were injected intraperitoneally with purified rat anti-mouse CD41 (25 μg in physiological saline). As a control, purified rat IgG1, κ isotype antibody (25 μg in physiological saline) was used. The efficiency of platelet depletion was confirmed as demonstrated previously by flow cytometry with an anti-mouse/rat CD42d–phycoerythrin (PE) antibody (eBioscience, San Diego, CA, USA) on a BD FACSVerse instrument [35].

### Femoral artery cannulation

In order to measure systemic blood pressure and heart rate, an intravenous catheter was inserted into the left femoral artery, as described previously [36]. Briefly, experimental animals were anesthetized with 2% isoflurane (in air supplied to a nose cone with 0.8 L/min flow rate) using precision anesthetic vaporizers during femoral artery catheterization and subsequently with intraperitoneally applied ketamine (100 mg/kg, Calypsol; Gedeon Richter, Budapest, Hungary) and xylazine (10 mg/kg, CP-Xylazine; CP-Pharma, Burgdorf, Germany) during blood pressure measurement. The depth of the anesthesia was frequently tested by checking the plantar nociception or corneal reflex, and additional anaesthetic was administered as necessary. The left femoral artery was cannulated under a stereomicroscope, and it was used for continuous systemic arterial pressure measurement. Body temperature was maintained between 36 and 37 °C throughout the experiment by using a heating pad, controlled by a rectal probe. Atipamezole was applied intraperitoneally (0.01 mg/kg; Sigma-Aldrich, St. Louis, MO, USA) to withdraw α2-agonistic effects of xylazine and to ensure a stable blood pressure throughout the experiment. Arterial blood pressure was measured and recorded continuously using the MP100 system and AcqKnowledge 3.72 software from Biopac Systems Inc. (Goleta, CA, USA).

### Infrared pulse oximetry

Hair on the right thigh of each mouse was removed using Veet gel (Unilever, UK). Oxygen saturation, heart rate, breath rate and breath distension were measured continuously using MouseOX pulse oximeter (Starr Life Sciences Corp., Oakmont, PA, USA) in accordance with the manufacturer's instructions, and recorded using the MP100 system and AcqKnowledge 3.72 software from Biopac Systems Inc. (Goleta, CA, USA).

### Introduction of controlled in vivo hypoxia

The controlled, stepwise induction of hypoxia was established by mixing pure oxygen, carbon-dioxide and nitrogen using a rotameter (P41A1-BA2, Aalborg Instruments & Controls Inc., Orangeburg, NY, USA) through a nose cone at 0.5 bar pressure. Flow rates were determined for each step in wild-type mice in a pilot experiment and were kept constant across each experimental group; the flow settings were as follows: I. Normoxia: 40 mL/min O_2_, 0 mL/min CO_2_, 70 mL/min N_2_. II. Mild Hypoxia: 30 mL/min O_2_, 0 mL/min CO_2_, 80 mL/min N_2_. III. Severe Hypoxia: 20 mL/min O_2_, 7 mL/min CO_2_, 100 mL/min N_2_. Arterial blood gas and pH measurements were performed throughout the experiments by a Radiometer ABL80 FLEX analyser (Bronshoj, Denmark).

### Data analysis of controlled in vivo hypoxia

Recorded parameters with the MP100 system and AcqKnowledge 3.72 software from Biopac Systems Inc. (Goleta, CA, USA) were resampled to 12.5 samples/sec, and the first 120 s after induction of each step of the ingravescent hypoxia for mean arterial pressure, oxygen saturation, heart rate and breath rate were evaluated and are presented as the mean ± SD. Acquired data points for 30 s interval (from 60 s until 90 s following the induction of the subsequent hypoxia step) were used to calculate statistical differences between experimental groups. The area under curve for the breath distension channel was calculated, where the baseline was set as the mean of the first 60 s during the initial normoxia step.

### Experimental design and statistical analyses

Sample size was calculated as described previously [37], and was estimated based on a pilot study in control mice. To adhere to the 3R reduction strategies, experimental mice were used in multiple experiments to reduce the number of experimental animals to the greatest possible extent. Specifically, following the initial set of experiments, surviving mice were randomly assigned to the next set of experiments. The exact number and origin of distinct biological samples taken without applying preselection criteria from experimental animals are indicated in their respective figures legend. In case of in vivo studies the dose selection was based on previous studies [23, 33, 35]. Statistical analysis was performed using the GraphPad Prism software v.6.07 from GraphPad Software Inc. (La Jolla, CA, USA). Values are presented as mean ± SEM, unless indicated otherwise; *n* represents the number of distinct biological samples in independent experiments. Box-and-whisker diagrams demonstrate statistical differences between control and treatment groups, where data shown are the median, the minimum and maximum values, and the interquartile range. Probability distribution of all continuous variables was performed; nonparametric data were analyzed using Kolmogorov–Smirnov test, whereas in case of normally distributed data, statistical analysis between two groups was performed with an unpaired two-tailed Student’s *t* test, while multiple group comparisons were analyzed with one-way ANOVA followed by Tukey’s *post-hoc* test, unless stated otherwise, and comparisons between multiple groups at different time points were performed using two-way ANOVA followed by Bonferroni’s *post-hoc* test. *Post-hoc* tests were conducted where the *F* value in the ANOVA reached a *p* value of less than 0.05, which was considered to be statistically significant. *Post-hoc* tests were considered statistically significant if *p* < 0.05.

## Results

### Type I glomus cells express P2Y12R, but not CB1R

**Fig. 1:**
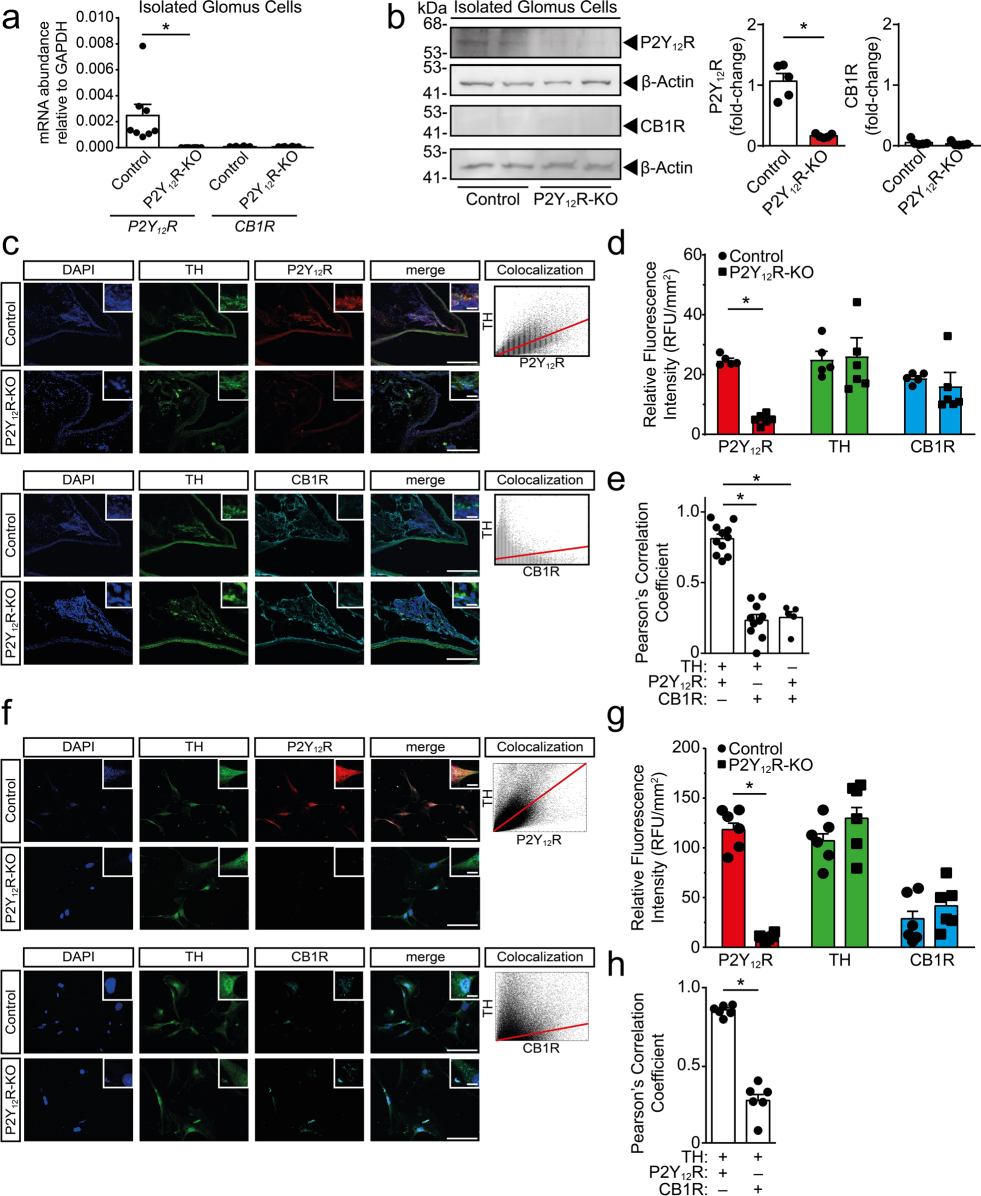
Glomus cells express P2Y12, but not CB1R receptors. **A**–**B** Expression of P2Y12R and CB1R were determined by RT-qPCR (**A**) and immunoblotting (**B**) on isolated glomus cell lysate in wild-type (Control) and P2Y12R-KO animals. Graphs show the relative expression of the indicated genes normalized to GAPDH (**A**) (n = 8) and densitometric evaluation of blots normalized to β-actin (**B**) (n = 5). **C**–**E** Representative immuno-confocal microscopy images of carotid body slices isolated from wild-type and P2Y12R-KO mice stained with antibodies directed against tyrosine-hydroxylase (TH, green), P2Y12R (red), CB1R (teal), nuclei (Hoechst 33,342, blue) and overlay image (merge). Scale bar: 100 μm, corresponds to 20 μm inset (**C**). Bar diagrams show the quantification fluorescence intensity (Control, n = 5; P2Y12R-KO, n = 6) (**D**), and the colocalization probability (n = 5–11) (**E**). **F**–**H** Representative immuno-confocal microscopy images of glomus cells in vitro isolated from wild-type and P2Y12R-KO mice stained with antibodies directed against tyrosine-hydroxylase (TH, green), P2Y12R (red), CB1R (teal), nuclei (Hoechst 33,342, blue) and overlay image (merge). Scale bar: 20 μm, corresponds to 10 μm inset (**F**). Quantification of fluorescence intensity (n = 6) (**G**), and the colocalization probability (n = 6) (**H**). Data represent the mean ± SEM; *, *p* ≤ 0.05 [one-way ANOVA with Tukey’s *post-hoc* test (**A**, **E**), unpaired two-tailed Student’s *t*-test (**B**, **H**), and two-way ANOVA with Bonferroni’s *post-hoc* test (**D**, **G**)]

Recent advances in the single cell transcriptome analysis of young, healthy mouse carotid body (CB) glomus cells (GC) have identified several highly expressed GPCRs, most interestingly P2Y purinoceptor 12 (P2Y12R) and Cannabinoid receptor 1 (CB1R) [21]. We have confirmed using RT-qPCR that isolated primary GCs from adult, healthy control mice express P2Y12R mRNA (Fig. 1A) (F [3, 28] = 7.991, p = 0.0005); additionally, P2Y12R protein was also present on the GC, but not on cells isolated from the P2Y12R-KO animals (Fig. 1B) (F [4, 4] = 77.15, P = 0.001). CB1R was undetected both on the mRNA (Fig. 1A) (F [3, 28] = 7.991, p > 0.9999), as well as on the protein level (Fig. 1B) (F [4, 4] = 2.234, P = 0.4053).

A specific feature of the GC is the synthesis and release of the neurotransmitter molecule dopamine, which process requires the presence of the tyrosine-hydroxylase (TH) enzyme [7]. Immunofluorescent labelling of TH, P2Y12R and CB1R molecules in CB slices demonstrated that TH and P2Y12R show with high probability the colocalization of the two molecules (Fig. 1C, E) (F [2, 23] = 83.42, p < 0.0001); whereas the colocalization probability of TH and CB1R (F [2, 23] = 83.42, p = 0.2649) or P2Y12R and CB1R is low (F [2, 23] = 83.42, p = 0.6282). The P2Y12R fluorescence intensity in the knock-out animals was absent (Fig. 1D) (F [1, 27] = 5.694, p < 0.0001). Additionally, we have tested the colocalization probability of TH, P2Y12R and CB1R proteins on isolated primary GC culture; similarly to the observed protein expression in CB slices, the colocalization probability between TH and P2Y12R was high, but was found to be modest between TH and CB1R (Fig. 1F–H) (F [5, 5] = 4.375, p < 0.0001). Based on our protein expression profile of the mature GC, the presence of the P2Y12R was confirmed, while the expression of CB1R protein could not be undoubtedly validated.

### Hypoxia and hypercapnia activates P2Y12R in primary GCs in vitro

**Fig. 2:**
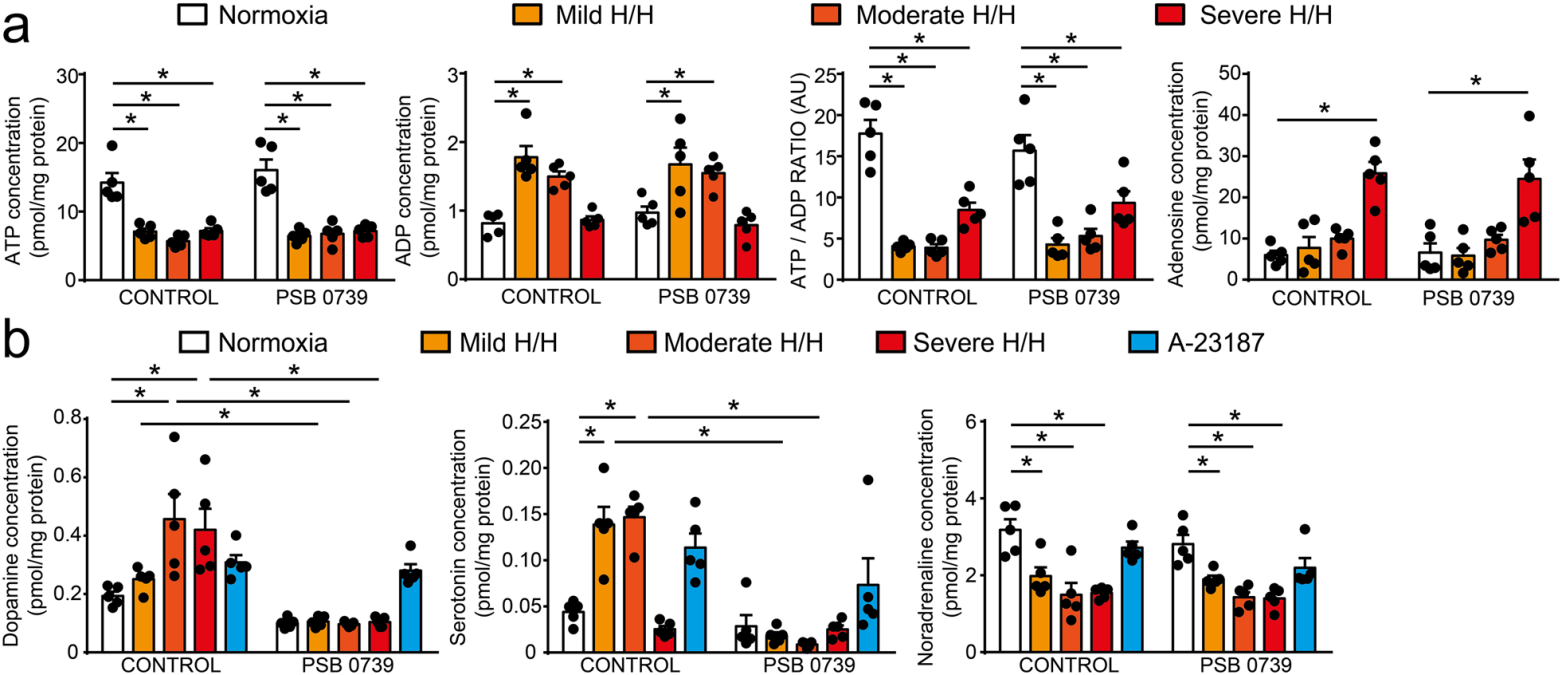
P2Y12R are required for hypoxia-induced monoamine release in vitro. **A**–**B** Bar diagrams show the quantification of ATP, ADP, adenosine concentration and the ratio of ATP/ADP levels measured from cell supernatant during H/H (ATP, n = 5; ADP, n = 5; ATP/ADP, n = 5; Adenosine, n = 5) (**A**), and the release of dopamine, noradrenaline and serotonin (DA, n = 5; 5-HT, n = 5; NA, n = 5) (**B**) in control and P2Y12R-inhibited (PSB 0739, 500 nM, 30 min.) cells cultures. Calcium-ionophore A-23187 (1 μM, 1 min) was used to evaluate the exocytosis of the reserve capacity of monoamines. Data represent the mean ± SEM; *, *p* ≤ 0.05 [two-way ANOVA with Bonferroni’s *post-hoc* test (**A**, **B**)]

Since our results establish the presence of P2Y12R on GC, but not that of CB1R, we have focused on the role of the purinergic system in the regulation of acute oxygen sensing and chemoreflex activation. Primary GCs were isolated and cultured in vitro from wild-type control mice, and cellular responses were studied during stepwise hypoxia and hypercapnia (H/H). Our initial results verified that H/H resulted in the reduction of extracellular ATP (F [3, 32] = 50.44, p < 0.0001), an increase in extracellular ADP concentration (F [3, 32] = 25.74, p = 0,0113), and a shift in the ATP/ADP ratio (Fig. 2A) (F [3, 32] = 50.18, p < 0.0001), which was closely followed by a substantial increase in extracellular adenosine concentration at the severe H/H stage (Fig. 2A) (F [3, 32] = 25.81, p < 0.0001). Administration of the specific P2Y12R inhibitor, PSB 0739, was without effect on the H/H-induced nucleotide changes (Fig. 2A) (ATP, F [3, 32] = 50.44, p > 0.9999; ADP, F [3, 32] = 25.74, p = 0.9999; ATP/ADP, F [3, 32] = 50.18, p = 0.9995; Adenosine, F [3, 32] = 25.81, p > 0.9999). Simultaneously, cultured GCs were activated during the stepwise H/H, and released neurotransmitters: a moderate level of H/H was sufficient to induce dopamine release (F [4, 40] = 5.835, p = 0.0006), and was completely abolished in the presence of PSB 0739 (Fig. 2B) (F [4, 40] = 5.835, p < 0.0001). Serotonin appeared in the cell culture medium during mild (F [4, 40] = 9.857, p = 0.0005) and moderate H/H (F [4, 40] = 9.857, p = 0.0001), but was absent during severe H/H; while the release was also blocked during P2Y12R inhibition (Fig. 2B) (F [4, 40] = 9.857, p = 0.0001). The stepwise increase in H/H resulted in the decrease of noradrenaline concentration (F [4, 40] = 21.05, p = 0.0057), but was unaffected by receptor blockade (Fig. 2B) (F [4, 40] = 21.05, p > 0.9999). Inhibition of P2Y12R under normoxic conditions had no effect on the observed changes in extracellular monoamine and nucleotide concentrations (Fig. 2A, B) (ATP, F [3, 32] = 50.18, p = 0.7852; ADP, F [3, 32] = 25.74, p = 0.9866; ATP/ADP, F [3, 32] = 50.18, p = 0.9064; Adenosine, F [3, 32] = 25.81, p > 0.9999; DA, F [4, 40] = 5.835, p = 0.7692; 5-HT, F [4, 40] = 9.857, p = 0.9978; NA, F [4, 40] = 21.05, p = 0.9527). Administration of the calcium-ionophore A-23187 induced the discharge of the remaining monoamine containing vesicles from GC; proving that the cells were still viable at the end of the stepwise H/H protocol and were able to release monoamines upon stimuli, corroborating the role of P2Y12R in the H/H-induced monoamine release (Fig. 2B) (DA, F [4, 40] = 5.835, p = 0.0429; 5-HT, F [4, 40] = 9.857, p = 0.0212; NA, F [4, 40] = 21.05, p = 0.0427).

### ADP-mediated P2Y12R activation modulates intracellular calcium levels and monoamine release in GCs in normoxic environment in vitro

**Fig. 3:**
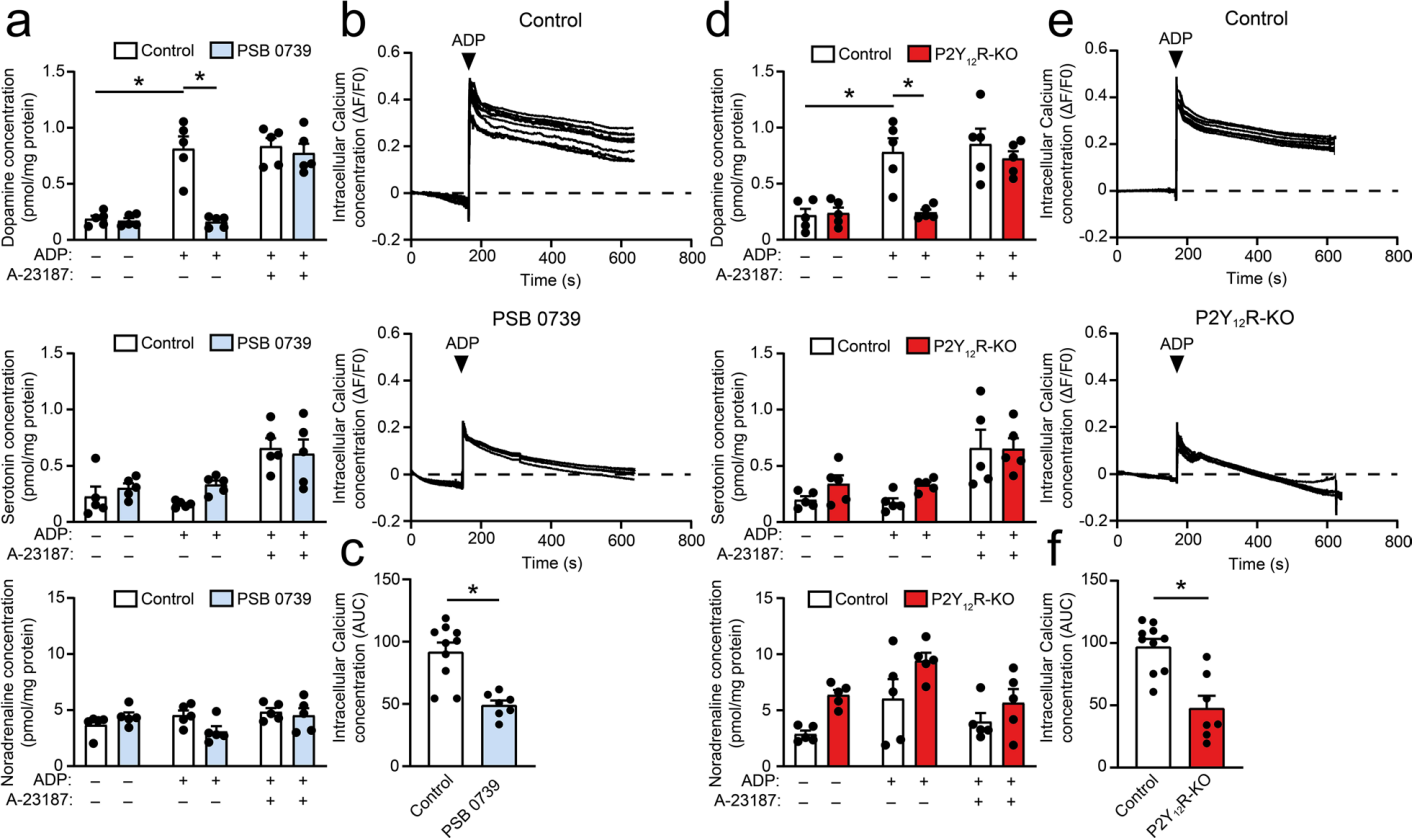
P2Y12R maintain intracellular calcium levels to facilitate dopamine release during hypoxia in vitro. **A**–**F** Monoamine release measured from cell supernatant after ADP (10 μM, 5 min) and A-23187 (1 μM, 1 min) administration in control, PSB 0739-treated (DA, n = 5; 5-HT, n = 5; NA, n = 5) (**A**) and P2Y12R-deficient cells (DA, n = 5; 5-HT, n = 5; NA, n = 5) (**D**). Representative graphs of the intracellular calcium concentration changes after ADP administration (10 μM, 5 min) in control, PSB 0739-treated (**B**) and P2Y12R-deficient cells (**E**). Intermittent lines illustrate the basal intracellular calcium levels prior to stimulation. Bar diagrams compare the area under curve (AUC) values of control vs. PSB 0739 (n = 7–10) (**C**) and wild-type vs. KO cells (n = 7–10) (**F**). Data represent the mean ± SEM; *, *p* ≤ 0.05 [two-way ANOVA with Bonferroni’s *post-hoc* test (**A**, **D**) and unpaired two-tailed Student’s *t*-test (**C**, **F**)]

Furthermore, activating the P2Y12R with the endogenous ligand ADP in the presence of the ectonucleotidase inhibitor ARL 67156 resulted in the release of dopamine (F [1, 24] = 20.5, p < 0.0001), but not that of serotonin (F [1, 24] = 1.164, p = 0.5502) or noradrenaline (F [1, 24] = 0.8257, p = 0.3197) (Fig. 3A, D). The effect of ADP was dependent on the presence of functional P2Y12R, since receptor inhibition or genetic modification completely abolished the ADP-induced dopamine release (Fig. 3A, D)(PSB 0739, F [1, 24] = 20.5, p = 0.9929; P2Y12R-KO, F [1, 24] = 9.324, p = 0.9981); however, treatment with A-23187 led to the release of dopamine and serotonin in receptor deficient cells (Fig. 3A, D) (PSB 0739, F [1, 24] = 20.5, p < 0.0001; P2Y12R-KO, F [1, 24] = 9.324, p = 0.0011). We have measured the effect of ADP stimulation on intracellular calcium concentration, where ADP treatment produced an initial calcium spike, followed by a lasting plateau phase (Fig. 3B, E). Receptor blockade and genetic deletion of P2Y12R resulted in a marked decrease in intracellular calcium levels during the plateau phase (Fig. 3C, F), which could contribute to the inefficient monoamine release during receptor stimulation (PSB 0739, F [6, 9] = 1.030, p < 0.0001; P2Y12R-KO, F [6, 9] = 3.878, p < 0.0001).

### The compensatory response to mild hypoxia requires adequate peripheral dopamine levels and is partly mediated by glomic P2Y12R in vivo

Since hypoxia and hypercapnia induced P2Y12R activation and receptor-dependent neurotransmitter release in isolated primary GCs, we further investigated the contribution of P2Y12R in the autonomic cardiorespiratory changes during hypoxia using an anaesthetized animal model. During the measurement, chemoreflex was activated by employing a transient, ingravescent hypoxia, and changes in mean arterial pressure (MAP), heart rate (HR), oxygen saturation, breath distension and respiratory rate were recorded.

**Fig. 4:**
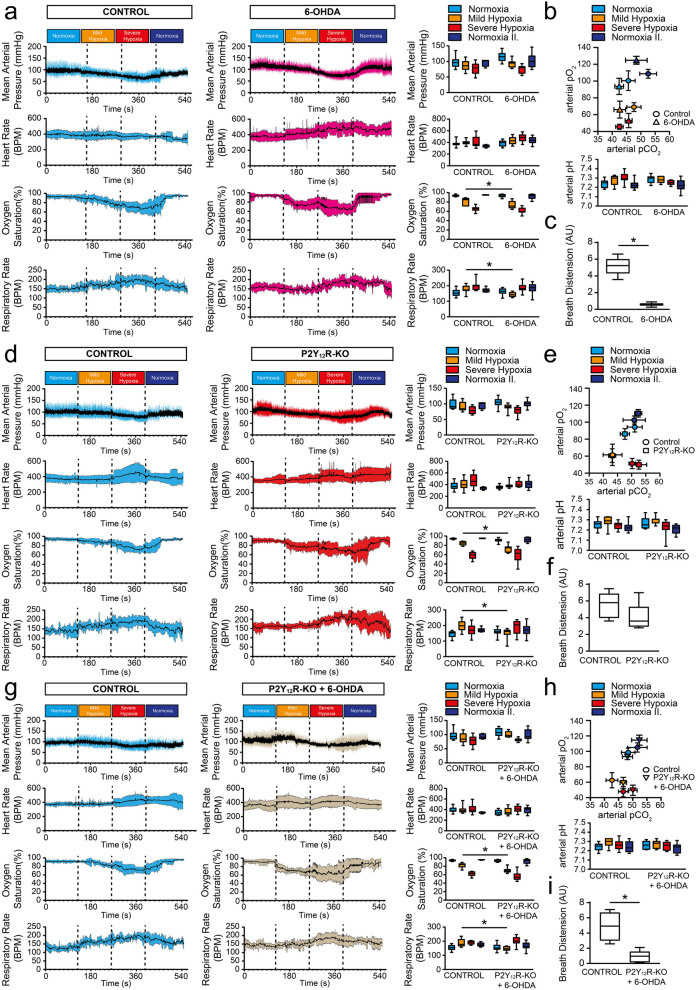
Ingravescent hypoxia stimulate chemoreflex via P2Y12R in vivo*.* **A**–**C** Experimental animals were treated with 6-OHDA (20 mg/kg i.p. for six alternative days) or the vehicle; and mean arterial pressure, heart rate, oxygen saturation and respiratory rate were recorded during ingravescent hypoxic challenge. Distinct stages of hypoxic environmental challenges are indicated with colors: normoxia (*light blue*), mild hypoxia (*yellow*) severe hypoxia (*red*) and recurrent normoxia (normoxia II., *dark blue*). Data represent the mean ± SD. Box-and-whisker diagrams demonstrate statistical differences between control and treatment groups during the identical challenge stage. Data show the median, the minimum and maximum values, and the interquartile range (n = 11) (**A**). Arterial blood-gas changes are shown for 6-OHDA compared to the respective control animals (n = 9; shown is the mean ± SD) and changes in arterial blood pH (n = 9) (**B**). Graphs compare the area under curve (AUC) values of breath distension in control and 6-OHDA mice (n = 5–8) (**C**). **D**–**I** P2Y12R-KO and littermate control animals were treated with 6-OHDA (**G**–**I**) or the vehicle (**D**–**F**); and mean arterial pressure, heart rate, oxygen saturation and respiratory rate were recorded during distinct stages of hypoxic environmental challenges. Diagrams demonstrate statistical differences between control, receptor-deficient and treatment groups during the equivalent challenge stage (n = 9–14 for P2Y12R-KO and n = 9–10 for 6-OHDA + P2Y12R-KO) (**D** and **G**). Arterial blood-gas and pH changes are shown for receptor-deficient (n = 11) (**E**) and receptor-deficient in combination with 6-OHDA-treatment (n = 9) (**H**) compared to the respective control animals (n = 9 for both control groups). Graphs compare the area under curve values of breath distension in the indicated experimental groups [n = 6 for P2Y12R-KO and n = 7 for 6-OHDA + P2Y12R-KO) (**F** and **I**). *, *p* ≤ 0.05 (two-way ANOVA with Bonferroni’s *post-hoc* test (**A**, **D**, **G**); one-way ANOVA with Tukey’s *post-hoc* test (B, E, H) and unpaired two-tailed Student’s *t*-test (**C**, **F**, **I**)]

Baseline physiological parameters of the particular control groups (Table 1) and between experimental groups were comparable (Table 2). In the control group, mild hypoxia, which effectively reduced the PaO_2_ and PaCO_2_ levels in the blood had a limited effect on MAP and HR; while oxygen saturation slightly, but significantly decreased and respiratory rate increased substantially (Fig. 4A, B). Subsequently, inducing severe hypoxia further reduced PaO_2_, without resulting in a PaCO_2_ elevation (Fig. 4B), markedly reduced MAP and O_2_ saturation, and considerably increased HR, respiration, and breath distension [i.e. the distension of the vascular bed caused by breathing, indicating that respiration was associated with larger fluctuations in the central venous pressure and cardiac output (Fig. 4A, C)]. 6-hydroxydopamine (6-OHDA) disrupts monoamine synthesis by producing reactive oxygen species, specifically destroys adrenergic nerve terminals and induces peripheral sympathectomy in experimental animals [38], effectively reducing base blood pressure and abolishing HR variation. In our experiments, a modified treatment protocol was used, where a low dose of 6-OHDA was administered intraperitoneally every 2 days for 2 weeks, which has been proven to effectively destroy adrenergic nerve terminals, without affecting baseline blood pressure and HR [34] (Table 2).

**Table 1:** Physiological parameters of control groups preceding hypoxic challenge

Normoxia						
	Control (littermate)	Control (6-OHDA)	Control (littermate/6-OHDA)	Control (Clopidogrel)	Control (PSB 0739)	Control (Platelet Depleted)
MAP (mmHg)	97.9 ± 16.7	98.8 ± 17.3	97.1 ± 16.9	104.4 ± 12.8	100.1 ± 12.0	108.2 ± 10.1
HR (BPM)	378.4 ± 68.6	375.9 ± 49.3	394.3 ± 65.3	382.1 ± 81.3	364.3 ± 59.3	416.3 ± 64.6
O_2_ Saturation (%)	94.4 ± 1.4	93.2 ± 1.9	93.3 ± 1.3	93.2 ± 1.6	92.8 ± 1.9	92.9 ± 2.1
RR (BPM)	144.5 ± 22.5	155.3 ± 23.1	150.9 ± 22.5	139.7 ± 12.9	134.3 ± 18.3	136.9 ± 8.9
PaO_2_ (mmHg)	100.4 ± 11.6	102.8 ± 9.8	98.7 ± 6.9	94.2 ± 8.8	94.1 ± 3.1	91.0 ± 10.1
PaCO_2_ (mmHg)	51.1 ± 2.9	45.5 ± 3.8	48.3 ± 6.7	49.7 ± 3.7	48.1 ± 1.4	46.9 ± 2.2
pH	7.24 ± 0.05	7.24 ± 0.04	7.25 ± 0.03	7.23 ± 0.03	7.24 ± 0.04	7.28 ± 0.06

*MAP* mean arterial pressure, *HR* heart Rate, *RR* respiratory rate, *PaO*_*2*_ arterial partial O_2_ pressure, *PaCO*_*2*_ arterial partial CO_2_ pressure

Data represent the mean ± SD. n.s., *p* ˃ 0.05 (one-way ANOVA with Tukey’s *post-hoc* test)

**Table 2:** Physiological parameters of experimental groups preceding hypoxic challenge

	Control (∑)	6-OHDA	P2Y12R-KO	P2Y12R-KO/6-OHDA	Clopidogrel	PSB 0739	Platelet depleted
MAP (mmHg)	98.4 ± 16.8	115.2 ± 15.7	104.9 ± 12.9	107.7 ± 14.9	102.1 ± 12.5	104.8 ± 10.0	102.2 ± 16.6
HR (BPM)	377.1 ± 57.1	385.9 ± 50.1	357.7 ± 29.1	344.7 ± 49.5	362.1 ± 33.9	381.2 ± 60.0	423.0 ± 66.2
O_2_ Saturation (%)	93.8 ± 1.7	93.2 ± 3.4	91.2 ± 3.6	93.0 ± 2.6	91.6 ± 3.9	92.9 ± 3.6	94.4 ± 2.4
RR (BPM)	150.5 ± 22.9	162.1 ± 21.2	160.3 ± 22.2	156.0 ± 25.3	154.2 ± 17.9	162.5 ± 40.7	150.0 ± 24.3
PaO_2_ (mmHg)	97.0 ± 16.1	93.6 ± 9.7	102.5 ± 4.8	93.1 ± 11.6	87.4 ± 6.8	87.6 ± 7.9	88.2 ± 3.7
PaCO_2_ (mmHg)	49.5 ± 8.2	41.8 ± 5.1	51.4 ± 4.4	48.1 ± 6.0	44.5 ± 7.9	41.7 ± 6.0	41.8 ± 4.8
pH	7.25 ± 0.04	7.28 ± 0.04	7.26 ± 0.05	7.26 ± 0.04	7.27 ± 0.06	7.28 ± 0.09	7.33 ± 0.06

*MAP* mean arterial pressure, *HR* heart Rate, *RR* respiratory rate, *PaO*_*2*_ arterial partial O_2_ pressure, *PaCO*_*2*_ arterial partial CO_2_ pressure

Data represent the mean ± SD. n.s., *p* ˃ 0.05 (one-way ANOVA with Tukey’s *post-hoc* test)

Nevertheless, we have also confirmed the removal of dopamine (F [5, 5] = 17.51, p = 0.0013) and the absence of TH-containing cells in the CB (F [8, 8] = 1.285, p = 0.0355), which disrupts the afferent signalling of oxygen sensing (Additional file 1: Figure S1A–C). 6-OHDA treatment was without effect on MAP and HR in comparison to the changes in the wild-type control animals; however, during mild hypoxia, the O_2_ saturation decreased markedly in treated mice, whilst the compensatory increase in respiratory rate was absent (Fig. 4A) (MAP, F [1, 67] = 3.030, p ≥ 0.9999; HR, F [1, 67] = 13.44, p = 0.7549; O_2_ Sat., F [1, 67] = 7.475, p = 0.0197; RR, F [1, 67] = 1.612, p = 0.0106). During severe hypoxia, 6-OHDA treated and control mice were indistinguishable, except that breath distension in the treated mice was greatly reduced, which might be due to the lack of sympathetic control on the vascular system (Fig. 4C) (MAP, F [1, 67] = 3.030, p =  > 0.9999; HR, F [1, 67] = 13.44, p = 0.0828; O_2_ Sat., F [1, 67] = 7.475, p = 0.6206; RR, F [1, 67] = 1.612, p > 0.9999).

Next, we investigated the involvement of P2Y12R in chemoreflex-induced cardiorespiratory changes, first by testing the genetically-modified P2Y12R-KO animals. Oxygen saturation and the increase in respiratory rate during mild hypoxia were significantly reduced in the knock-out mice (Fig. 4D) (O_2_ Sat., F [1, 81] = 5.068, p = 0.0092; RR, F [1, 81] = 0.05696, p = 0.0075), although there was no difference in the PaO_2_ and PaCO_2_ levels or blood pH (Fig. 4E) (pO2 F [1, 69] = 1.183, p = 0.4860; pCO2 F [1, 69] = 0.000395, p = 0.7453; pH F [1, 79] = 0,06262, p = 0.9983). Likewise, receptor-deficiency did not alter MAP and HR changes (Fig. 4D) (MAP, F [1, 81] = 1.041, p > 0.9999; HR, F [1, 81] = 0.37843, p > 0.9999) or breath distension (Fig. 4F) (F [4, 5] = 1.075, p = 0.1780) during ingravescent hypoxic challenge. Subsequent chemical sympathectomy (6-OHDA administration) in the P2Y12R-KO animals demonstrated a marked reduction in oxygen saturation and an absence of the compensatory increase in breath rate in mild hypoxic environment; whilst changes in MAP and HR, as well as arterial blood gas parameters were similar to the control animals (Fig. 4G, H) (MAP, F [1, 65] = 5.126, p > 0.9999; HR, F [1, 65] = 0.3816, p > 0.9999; O2 Sat., F [1, 65] = 15.79, p = 0.0002; RR, F [1, 65] = 2.492, p = 0.0245). During severe hypoxia, the 6-OHDA-treated knock-out mice displayed identical cardiorespiratory and blood gas changes as the littermate control mice (Fig. 4G, H) (MAP, F [1, 65] = 5.126, p > 0.9999; HR, F [1, 65] = 0.3816, p > 0.9999; O2 Sat., F [1, 65] = 15.79, p = 0.9057; RR, F [1, 65] = 2.492, p = 0.9312; pO_2_ F [1, 62] = 0.04002, p > 0.9999; pCO_2_ F [1, 62] = 0.004785, p > 0.9999; pH F [1, 63] = 0,4739, p = 0.9849); whereas breath distension in the treated, receptor-deficient mice was markedly reduced (Fig. 4I) (F [3, 6] = 7.294, p = 0.0007).

**Fig. 5:**
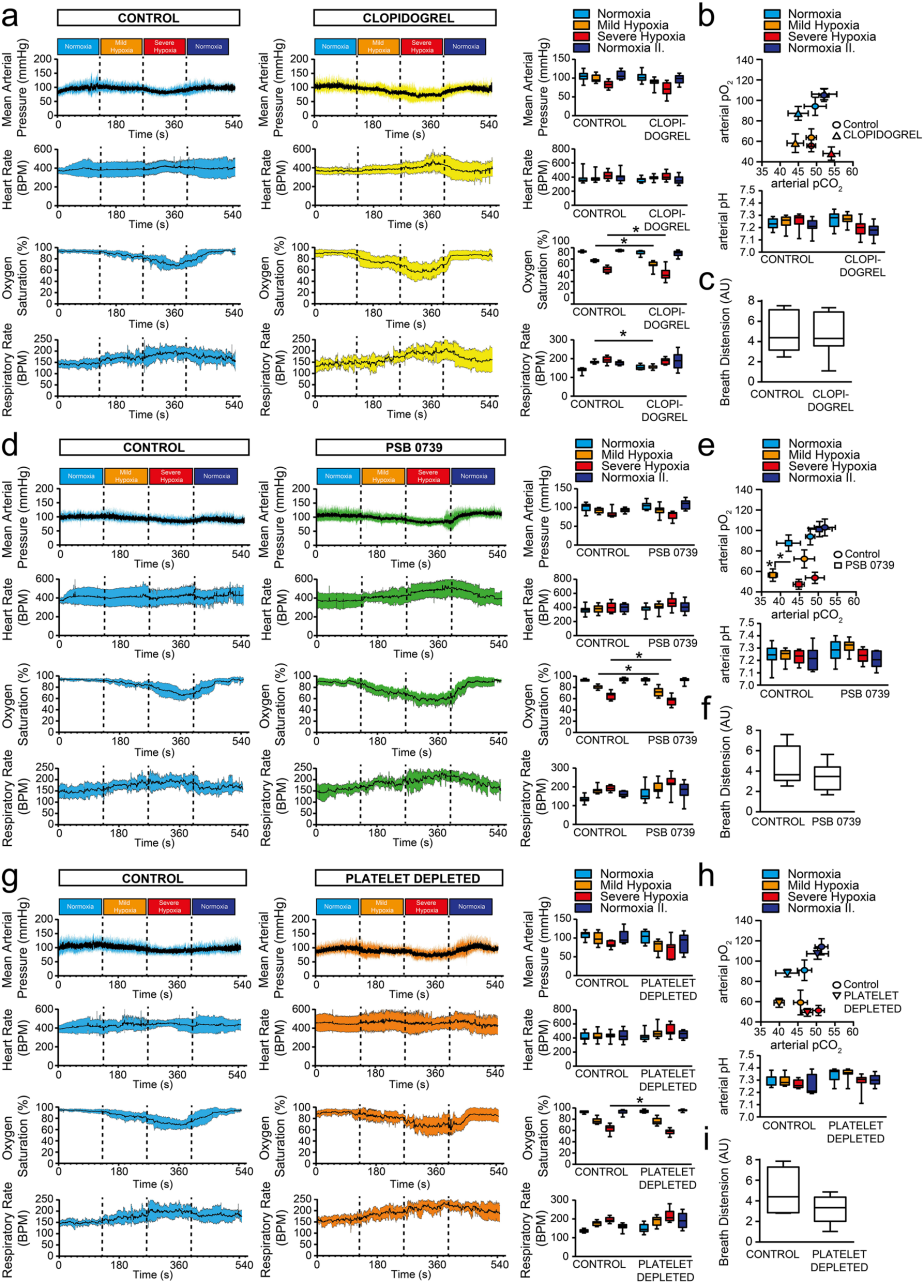
Glomic P2Y12R, but not centrally expressed receptors or thrombocytes influence chemoreflex in vivo. **A**–**I** Experimental animals were treated with clopidogrel (10 mg/kg i.p., 60 min prior to the experiment) (**A**–**C**); PSB 0739 (0.3 mg/kg i.t., 18 h prior to the experiment) (**D**–**F**); the mouse specific anti-CD41 antibody (deplete thrombocytes) (**G**–**I**) or the respective vehicle; and mean arterial pressure, heart rate, oxygen saturation and respiratory rate were recorded during ingravescent hypoxic challenge. Distinct stages of hypoxic environmental challenges are indicated with colors: normoxia (*light blue*), mild hypoxia (*yellow*) severe hypoxia (*red*) and recurrent normoxia (normoxia II., *dark blue*). Data represent the mean ± SD. Box-and-whisker diagrams demonstrate statistical differences between control and treatment groups during the identical challenge stage. Data show the median, the minimum and maximum values, and the interquartile range (n = 11 for Clopidogrel; n = 12 for PSB 0739; and n = 10 for platelet depleted) (**A**, **D**, **G**). Arterial blood-gas and pH changes are shown for clopidogrel (n = 9) (B), PSB 0739 (n = 8) (**E**) and platelet depleted groups (n = 10) (**H**) compared to the respective control animals (n = 9 for Control (clopidogrel); n = 12 Control (PSB 0739); and n = 10 Control (depleted) groups). Graphs compare the area under curve (AUC) values of breath distension for the clopidogrel (n = 7) (**F**) and platelet depleted experimental groups (n = 5–8) (**I**). *, *p* ≤ 0.05 [two-way ANOVA with Bonferroni’s *post-hoc* test (**A**, **D**, **G**); one-way ANOVA with Tukey’s *post-hoc* test (**B**, **E**, **H**) and unpaired two-tailed Student’s *t*-test (**C**, **F**, **I**)]

Following, we have evaluated the effect of acute P2Y12R inhibition, by administering a single dose clopidogrel intraperitoneally. Clopidogrel is metabolized in the liver to form an active metabolite, and is unable to cross the blood brain barrier, effectively acting only on peripherally located P2Y12R [39]. Clopidogrel-treatment considerably and significantly reduced oxygen saturation and abrogated the compensatory increase in breath rate during mild hypoxia, and further reduced oxygen saturation during severe hypoxia, while receptor blockade had a limited effect on MAP and HR changes (Fig. 5A) (MAP, F [1, 72] = 9.607, p = 0.1606; HR, F [1, 72] = 1.841, p > 0.9999; O2 Sat., F [1, 72] = 17.2, p = 0.0254; RR, F [1, 72] = 0.1466, p = 0.0491). Furthermore, treated mice demonstrated hypercapnia during severe hypoxia, although the increase in PaCO_2_ level did not reach statistical significance (Fig. 5B) (pCO2 F [1, 68] = 2.288, p = 0.7157). Breath distension parameters were identical in both experimental groups (Fig. 5C) (F [4, 6] = 1.774, p = 0.4896).

Recent work has demonstrated the role of microglia in neurovascular coupling to regulate the function of respiratory centres in the medulla oblongata, primarily during hypercapnic conditions [27]. Since P2Y12R are also present in the central nervous system (CNS), where the receptor is expressed preferentially by microglia, we have administered the selective P2Y12R-blocker PSB 0739 intrathecally. The compound is impermeable to the blood brain barrier, implicating that centrally expressed receptors are selectively inhibited, while receptors expressed on peripheral tissues are unaffected. PSB 0739-treatment was without effect on cardiovascular parameters; however, significantly reduced O_2_ saturation during mild and severe hypoxia, without influencing the compensatory increase in the respiratory rate (Fig. 5D) (MAP, F [1, 72] = 2.753, p > 0.9999; HR, F [1, 72] = 4.317, p > 0.9999; O_2_ Sat., F [1, 72] = 13.13, p = 0.0023; RR, F [1, 72] = 8.726, p > 0.9999). Moreover, arterial pO_2_ was considerably lower during mild hypoxic conditions in PSB 0739-treated animals, while PaCO_2_ level demonstrated hypocapnia compared to control mice (Fig. 5E) (pO2 F [1, 56] = 17.42, p = 0.0003; pCO2 F [1, 68] = 10.91, p = 0.1953). The changes in blood gas parameters were indistinguishable between the treated and control groups during severe hypoxia (Fig. 5E) (pO_2_ F [1, 56] = 17.42, p = 0.3842; pCO_2_ F [1, 68] = 10.91, p > 0.9999; pH F [1, 68] = 3.307, p = 0.0600). Additionally, no difference could be measured in breath distension after PSB 0739 administration during hypoxic challenge (Fig. 5F) (F [6, 6] = 2.101, p = 0.3700).

Finally, since P2Y12Rs are not exclusively expressed on the carotid body glomus cells, but are also present on platelets in the periphery, we have tested the potential involvement of thrombocytes in oxygen regulation and chemoreflex activation. The anti-CD41 (αIIb) monoclonal antibody was administered to deplete platelets in the experimental animals [40], and ingravescent hypoxia challenge was performed. Thrombocyte depletion had limited effect on cardiovascular parameters, oxygen saturation, respiratory rate or blood gas changes or breath distension during the experiment (Fig. 5G–I) (MAP, F [1, 72] = 11.15, p = 0.4958; HR, F [1, 72] = 5.996, p = 0.6004; O2 Sat., F [1, 72] = 0.3779, p > 0.9999; RR, F [1, 72] = 15.08, p = 0.7772; pO2 F [1, 67] = 2.113, p > 0.9999; pCO2 F [1, 67] = 21.07, p = 0.1583; pH F [1, 67] = 13.39, p = 0.0943; BD F [4, 7] = 4.103, p = 0.3245), suggesting that thrombocytes have minimal role in the chemoreflex activation in anaesthetized mice.

## Discussion

The carotid body constantly surveys and detects variations in the composition of the arterial blood, *e.g.* the partial pressure of arterial oxygen (PaO_2_), carbon dioxide (PaCO_2_), pH, but also the concentration of circulating metabolites and glucose [41]. In response to these changes, released neurotransmitters trigger action potential through the afferent fibres of the carotid sinus nerve, relaying information to the central nervous system to control cardiorespiratory homeostasis [42].

Peripheral chemoreceptors sense and transduce hypoxia through different mechanisms converging on the principal intracellular pathway, where elevated intracellular Ca^2+^ concentration leads to neurotransmitter secretion [43]. Apparently, Ca^2+^ is almost exclusively derived from extracellular sources, entering the cell via voltage-dependent Ca^2+^ channels, induced by the inhibition of K^+^ channels and the subsequent membrane depolarization [42]. Furthermore, circulating metabolites and GPCRs are also involved in chemoreflex [18, 44]. The various stimuli affecting oxygen sensing in the CB suggest that instead of a single sensor, a wide range of sensors with different affinities and thresholds elicit the adequate response [16]. The principal new findings of this study is establishing the presence of glomic P2Y12Rs, and the role of the receptor during hypoxia-induced monoamine transmitter release via regulating intracellular calcium levels; proposing a novel mechanism to sufficiently compensate cardiorespiratory changes during moderate hypoxia.

The G_i_-coupled purinergic P2Y_12_-receptor is sensitive to changes in the levels of nucleotides, primarily extracellular ADP. In peripheral tissues, P2Y12Rs are present on platelets [45]; while centrally, microglia express the receptor [46]. Additionally, preliminary results also suggest that both TH positive and negative cells isolated from CB show P2Y12R expression [22]. The G_i_-coupled cannabinoid-1 receptor, activated by endocannabinoids, such as anandamide and 2-AG, is widely distributed and functions as an important signal modulator in most tissues [47]. Based on the published expression-profiling data of murine glomus cells [21], we have demonstrated that P2Y12R are present on TH-expressing type I glomus cells, but not other cell types in the CB; however, the expression of CB1R could not be validated. A potential explanation for the observed difference between the sequencing data of the healthy, young murine GC and our results studying the RNA and protein level of CB1R in mature GC might be that either CB1R receptors show age-related transient expression [48, 49]. Alternatively, it has been proven that the transcription and translation of CB1R occur in response to prolonged pathophysiological environment [50, 51]. Nevertheless, we were able to conclusively validate the expression of only P2Y12R, but not CB1R in the mouse carotid body glomus cell.

To understand the function of P2Y12R in the regulation of acute oxygen sensing, the release of purinergic neurotransmitters was analysed during hypoxia and hypercapnia. During controlled, stepwise H/H, the concentration of extracellular ATP markedly decreased, while ADP level increased significantly, inducing a shift in the ATP/ADP ratio. Furthermore, the concentration of adenosine increased considerably. The increased availability of purinergic mediators in hypoxic environment may indicate that the ligands and respective receptors might contribute to chemotransduction.

Since GCs express TH enzyme and produce and release monoamine transmitters during stimulation [7], we measured the concentration of the released monoamine neurotransmitters during H/H. The extracellular concentration of dopamine and serotonin significantly increased, and the observed changes were dependent on the presence of functional P2Y12R.

Considering that in the hypoxic environment, the ATP/ADP ratio is reduced, as well as that dopamine and serotonin release was dependent on P2Y12R, we next investigated whether ADP-induced P2Y12R activation is able to mimic the monoamine release observed during H/H. ADP administration in the presence of an ectonucleotidase inhibitor, which prevents rapid metabolism to adenosine, promoted the release of dopamine, but not that of serotonin or noradrenaline. Furthermore, P2Y12R inhibition or genetic modification completely abolished the ADP-induced dopamine release. Based on our results, we presume that the shift in the ATP/ADP ratio in hypoxic environment is a sufficient signal to stimulate GC, activate P2Y12R and induce monoamine discharge in vitro.

Platelets react to bleeding from vascular injury by thrombus formation. Activation of P2Y12R is pivotal for platelet activation, adhesion, thrombus growth and stability [52]. It has been shown that while the receptor does not induce intracellular calcium mobilization [53], it maintains intracellular calcium levels when G_q_ or G_12/13_ pathways are stimulated [54]. P2Y12R may fulfil a similar role in GC, as observed in thrombocytes: receptor activation with ADP produced an initial calcium spike, followed by a lasting plateau phase. Impaired P2Y12R function tempered the increment of the initial Ca^2+^ spike and abrogated the plateau phase, which could potentially be responsible for the insufficient monoamine release. Albeit the origin of the initial Ca^2+^ stimulus thus far is uncertain, it is highly likely that other purinergic receptors may partake in chemotransduction; the Gα_q/11_-coupled P2Y_1_-receptors has been shown to respond to ADP stimulation and regulate intracellular Ca^2+^ concentration in glomus cells [55]. Nevertheless, to conclusively establish the exact nature of the initial Ca^2+^ spike necessitates further experimental work.

Lastly, based on our in vitro findings, we have explored the function of P2Y12R on the autonomic cardiorespiratory changes during hypoxia in vivo using an anaesthetized animal model. To test the effect of the hypoxic environment, a stepwise, ingravescent hypoxia model in healthy, wild-type control mice was established, which markedly reduced MAP and increased HR, significantly reduced O_2_ saturation and PaO_2_, without affecting PaCO_2_ levels. To counteract the detrimental effect of the hypoxic condition, a compensatory increase appeared in breathing rate accompanied by elevated breath distension. Breath distension reflects changes in the blood volume in the peripheral vascular bed that correspond to regional blood pressure fluctuations generated by increased breathing effort [36]. Notably, in the control animals, a moderate degree of PaO_2_ reduction has already maximally stimulated the compensatory increase in respiration to mitigate the depression in O_2_ saturation; whereas during severe hypoxia, since the reserve capacity of breathing rate was exhausted, most likely the degree of breath depth was involved in the compensatory response, as indicated by the increase in breath distension.

Since in vitro, the primary GC activation resulted in a marked increase in extracellular dopamine concentration, to assess the involvement of monoamines in the chemoreflex activation in our experimental model, peripheral sympathetic denervation was employed to eliminate dopamine in peripheral tissues. Peripheral removal of monoamines abolished the compensatory increase in respiratory rate and O_2_ saturation decreased sharply during mild hypoxic challenge, while during severe hypoxia, comparable cardiorespiratory responses were observed. Interestingly, breath distension was absent in the treated mice, which could either be the consequence of the loss of the sympathetic nerve regulation in resistance vessels or potentially due to the insufficiency of the compensation by adjusting tidal volume.

Considering that dopamine release required the presence of functional P2Y12Rs, we have evaluated the effect of genetic deletion of P2Y12R using a constitutive knock-out experimental mouse model. Receptor-deficiency produced a significant reduction in breath rate and a pronounced decrease in O_2_ saturation during mild hypoxia, while there was no difference in cardiorespiratory or blood gas parameters during severe hypoxic challenge. Additionally, chemical sympathectomy in P2Y12R-KO animals exhibited comparable changes to 6-OHDA-treated wild-type and P2Y12R-KO animals during the hypoxic challenge. This observation abrogates the prospect of an additive or synergistic relationship between P2Y12R-induced— and catecholamine-dependent cardiorespiratory compensatory responses during hypoxia; consequently may indicate that the receptor stimulation and catecholamine secretion from GC during chemoreflex activation occur sequentially.

Due to the constitutive nature of the genetically-modified strain, where modulation of compensatory pathways potentially occurs, we have tested pharmacological tools to acutely block P2Y12R function. In the clinical practice, the widely-used antithrombotic agent, clopidogrel, is a specific, irreversible P2Y12R inhibitor [56]. Notably, clopidogrel is a prodrug that undergoes metabolism in the liver to form the active metabolite [57], where the active metabolites have a low blood–brain barrier (BBB) permeability [39]. Intraperitoneal administration of clopidogrel, which principally effected receptors located on thrombocytes and GC, markedly suppressed compensatory respiratory rate increase during mild hypoxia, consequently O_2_ saturation also decreased considerably; moreover, during severe hypoxic challenge, clopidogrel treated mice demonstrated decreased oxygen saturation. These observations may indicate that peripherally expressed P2Y12R engages in chemoreflex activation and influences the compensatory cardiorespiratory changes.

To assess the involvement of the centrally expressed receptors, the effect of intrathecally administered P2Y12R antagonist, which is unable to cross the BBB [32], was investigated. Central receptor inhibition was without effect on the compensatory breath rate increase, while decidedly reduced oxygen saturation and blood gases during hypoxic challenge. Recently, microglial P2Y12Rs have been proven to interact in neurovascular coupling and regulate hypercapnia-induced vasodilatation and cerebral blood flow increase [27]. Interestingly, central inhibition of the receptor function was without effect on the compensatory breath rate increase during ingravescent hypoxia, potentially implying that distinct signalling circuits are present during chemoreflex activation; and the compensatory response in respiration rate occurs independently of the microglial receptors.

Ultimately, the potential influence of platelet expressed P2Y12R on the chemoreflex activation was explored by depleting thrombocytes with the mouse-specific anti-CD41 antibody. Platelet-depleted mice demonstrated similar cardiorespiratory and blood gas changes during hypoxia to the respective control group; excluding the involvement of thrombocytes in the cardiopulmonary compensatory mechanism.

Recently, Tubek and colleagues published a prospective patient pilot study [58], where the hypoxic and hypercapnic ventilatory responses were compared of peripherally acting P2Y12R-inhibitors in eleven patients undergoing percutaneous coronary intervention. They have found that four weeks of clopidogrel-treatment had no effect on hypoxic responses, whereas a switch from clopidogrel to ticagrelor conversely increased ventilatory responses to intermittent hypoxia and hypercapnia. It is important to note that all patients received concomitant medication, that have been shown to influence the chemoreflex arc [59]; furthermore, isocapnia was not maintained during hypoxic measurements. Nonetheless, it is intriguing that the increase in ventilatory responses observed after switching medication to ticagrelor may suggest a reduction in the tonic inhibitory effect of clopidogrel on the chemosensory apparatus. While the precise effect of peripherally acting P2Y12R-inhibitors on chemoreceptor sensitivity is still unclear, it is compelling to further investigate the translational aspect of glomic P2Y12R function; potentially involving patients with chronic respiratory morbidities.

## Conclusions

**Fig. 6:**
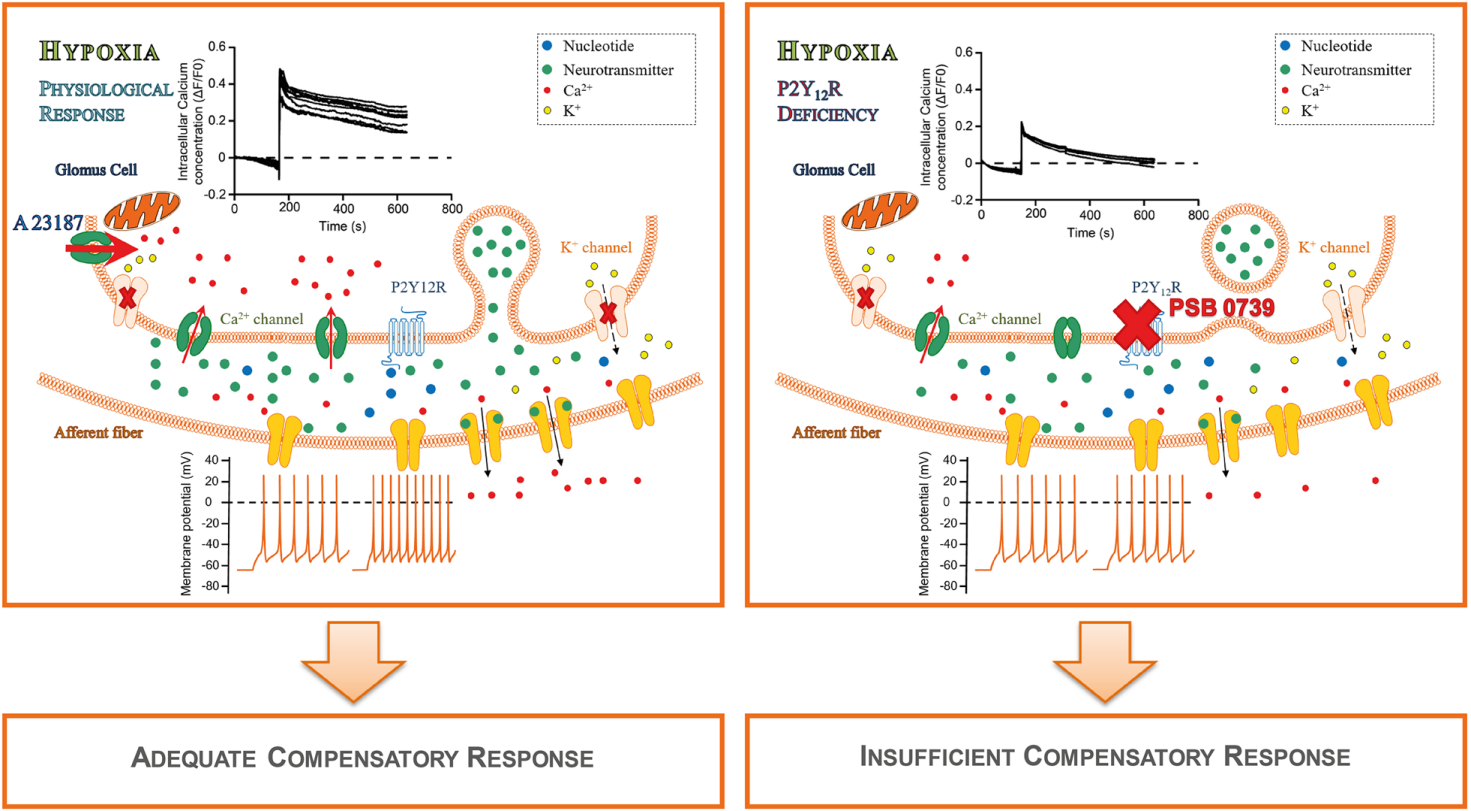
Putative model of glomus cell P2Y12-receptor and its downstream signalling mechanism during hypoxia. In a hypoxic environment, the concentration of extracellular nucleotides increases, leading to the activation of P2Y12Rs. P2Y12R in turn facilitates the increase in intracellular calcium levels and the persistence of a plateau phase essential to evoke monoamine release and for the manifestation of adequate cardiovascular compensatory responses. Lack of the receptor function, either via genetic-deficiency or pharmacological agents (PSB 0739), abrogates hypoxia-induced receptor activation, diminishes the intracellular calcium concentration, and leads to insufficient monoamine release; resulting in impaired cardiovascular compensatory changes to hypoxia

A principal function of the carotid body is the surveillance of the oxygenation state of the circulating blood in order to guide cardiorespiratory centres to provide sufficient oxygen to tissues. Our results demonstrate that P2Y12R expressed on GC are required for hypoxia-induced monoamine transmitter release via the receptor mediated sustained increase in intracellular calcium levels, in order to sufficiently compensate during moderate hypoxia. However, during severe hypoxic conditions, peripheral mechanisms have limited effect and most likely central mechanisms are involved in the compensatory response in anaesthetised animals. Importantly, considering that P2Y12R are present on glomus cells and have a fundamental role in chemoreflex activation already during moderate arterial pO_2_ reduction, which can be attributed to respiratory diseases, such as COPD [60]; antiplatelet medication may inadvertently influence peripheral chemoreflex sensitivity, consequently the clinical treatment benefit might be reduced for patients with chronic respiratory morbidities (Fig. 6).

### Supplementary Information

**Additional file 1: Figure S1.** Validation of 6-OHDA treatment efficiency in mice. (A) Representative immuno-confocal microscopy images of Carotid Body slices isolated from wild-type and 6-OHDA treated (20 mg/kg 6-OHDA i.p. for six alternative days) mice stained with antibodies directed against tyrosine-hydroxylase (TH, green), P2Y12R (red), nuclei (Hoechst 33342, blue) and overlay image (merge). Scale bar: 100 μm. Bar diagrams show the quantification of TH and P2Y12R fluorescence intensity (n = 9 for TH; n = 9–12 for P2Y12R). (B-C) Bar diagrams show the quantification of dopamine in whole Carotid Body tissue lysates (n = 6) (B) and plasma (n = 6) (C) in wild-type control and 6-OHDA treated (20 mg/kg 6-OHDA for six alternative days) mice. Data represent the mean ± SEM; *, p ≤ 0.05 (unpaired two-tailed Student’s t-test (A-C)).

## Data Availability

All data supporting the findings of this study are available within the paper and it’s Supplementary Information. All other information that supports the findings of this study is available from the corresponding author upon reasonable request.

## Abbreviations

ADP: Adenosine diphosphate
2-AG: 2-Arachidonoylglycerol
ANOVA: Analysis of variance
ANS: Autonomic nervous system
ATP: Adenosine triphosphate
BK: Big potassium
CB: Carotid body
CB1R: Cannabinoid receptor 1
CNS: Central nervous system
COPD: Chronic obstructive pulmonary disease
DA: Dopamine
DBH: Dopamine-β-hydroxylase
GABA: γ-Aminobutyric acid
GC: Glomus cell
GPCR: G-protein coupled receptor
H/H: Hypoxia and hypercapnia
HR: Heart rate
KO: Knock-out
MAP: Mean arterial pressure
NA: Noradrenaline
NADH: Nicotinamide adenine dinucleotide
P2Y12R: P2Y_12_-receptor
PaCO_2_: Partial pressure of arterial carbon dioxide
PaO_2_: Partial pressure of arterial oxygen
PBS: Phosphate buffered saline
pH: Potential of hydrogen
RR: Respiratory rate
SD: Standard deviation
SEM: Standard error of mean
TASK: TWIK-related acid-sensitive K^+^
TH: Tyrosine hydroxylase
WT: Wild-type
