# Supplementary material for: Blood oxygen regulation via P2Y12R expressed in the carotid body
Source: Respir Res. 2024 Jan 27;25:61. doi: 10.1186/s12931-024-02680-x (PMC10821555; doi:10.1186/s12931-024-02680-x)
Supplement: Supplementary file 1 — Additional file 1: Figure S1. Validation of 6-OHDA treatment efficiency in mice. (A) Representative immuno-confocal microscopy images of Carotid Body slices isolated from wild-type and 6-OHDA treated (20 mg/kg 6-OHDA i.p. for six alternative days) mice stained with antibodies directed against tyrosine-hydroxylase (TH, green), P2Y12R (red), nuclei (Hoechst 33342, blue) and overlay image (merge). Scale bar: 100 μm. Bar diagrams show the quantification of TH and P2Y12R fluorescence intensity (n = 9 for TH; n = 9–12 for P2Y12R). (B-C) Bar diagrams show the quantification of dopamine in whole Carotid Body tissue lysates (n = 6) (B) and plasma (n = 6) (C) in wild-type control and 6-OHDA treated (20 mg/kg 6-OHDA for six alternative days) mice. Data represent the mean ± SEM; *, p ≤ 0.05 (unpaired two-tailed Student’s t-test (A-C)). [file 12931_2024_2680_MOESM1_ESM.docx]

1. **Additional Data**


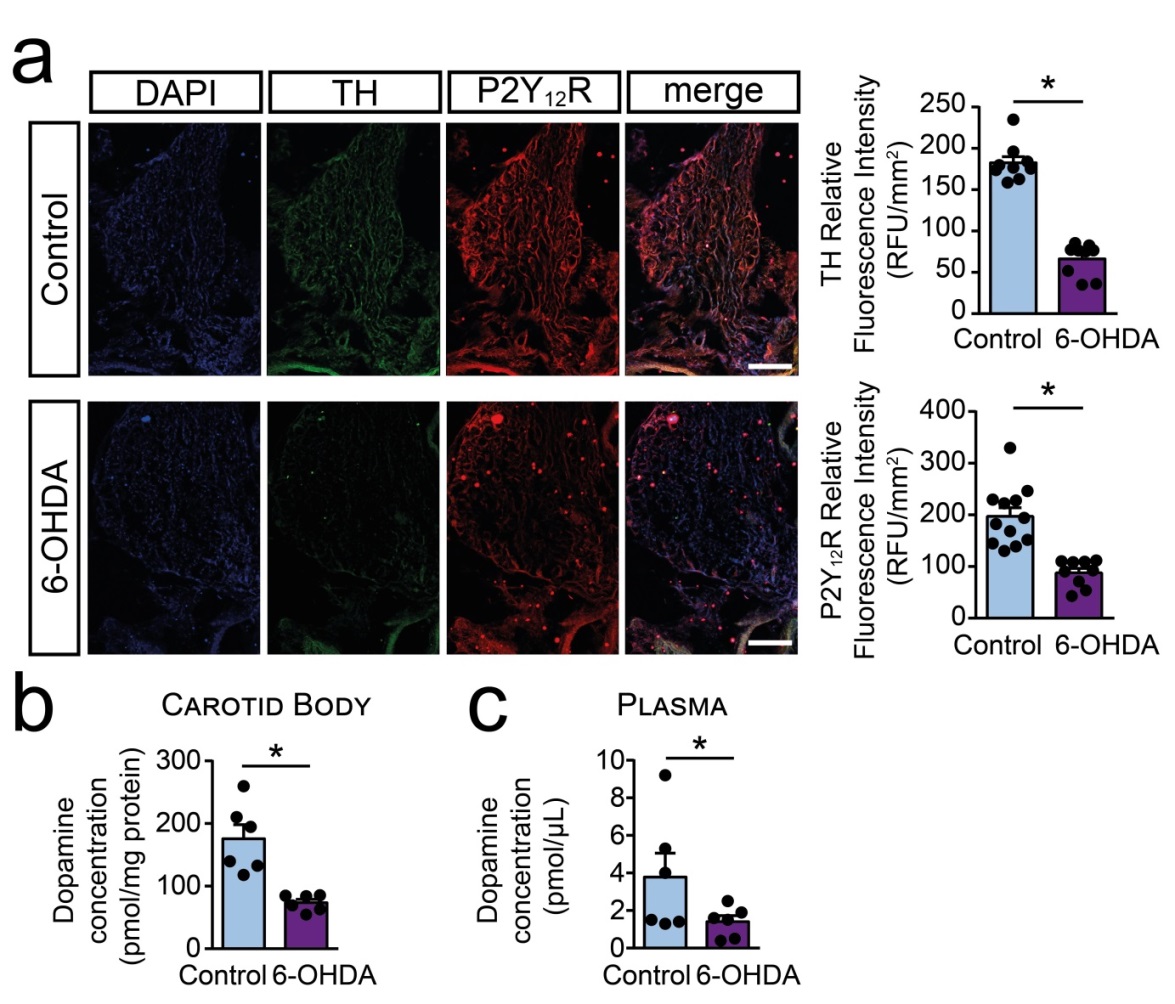


**Additional Figure 1. Validation of 6-OHDA treatment efficiency in mice**

**(A)** Representative immuno-confocal microscopy images of Carotid Body slices isolated from wild-type and 6-OHDA treated (20 mg/kg 6‑OHDA i.p. for six alternative days) mice stained with antibodies directed against tyrosine-hydroxylase (TH, green), P2Y12R (red), nuclei (Hoechst 33342, blue) and overlay image (merge). Scale bar: 100 μm. Bar diagrams show the quantification of TH and P2Y12R fluorescence intensity (n=9 for TH; n=9-12 for P2Y12R). **(B-C)** Bar diagrams show the quantification of dopamine in whole Carotid Body tissue lysates (n=6) (B) and plasma (n=6) (C) in wild-type control and 6-OHDA treated (20 mg/kg 6‑OHDA for six alternative days) mice. Data represent the mean ± SEM; *, *p* ≤ 0.05 (unpaired two-tailed Student’s *t*-test (A-C)).
